# The Impact of Caregiving Intensity and Financial Burden on the Health-Related Quality of Life Among Informal Caregivers for Patients With Advanced Lung Cancer: A Multicenter Cross-Sectional Study

**DOI:** 10.1155/jonm/8847157

**Published:** 2025-10-30

**Authors:** Yi Yang, Qingwen Deng, Liu Liu, Yingyao Chen

**Affiliations:** ^1^School of Public Health, Fudan University, Shanghai, China; ^2^National Health Commission Key Laboratory of Health Technology Assessment (Fudan University), Shanghai, China

## Abstract

**Aims:**

To determine the relationship between caregiving burden (including caregiving intensity and financial burden) and health-related quality of life (HRQoL) of informal caregivers, as well as to explore their association with the HRQoL of patients with advanced lung cancer.

**Background:**

Against the backdrop of aging and high prevalence of tumor disease, caregiving support for cancer patients is mostly provided by informal caregivers. The caregiving burden resulting from this support may have adverse effects on the health of informal caregivers. Few studies have examined these effects simultaneously in relation to caregiving burden.

**Methods:**

This hospital-based cross-sectional study collected questionnaire data through face-to-face interviews at 13 centers in China. A total of 540 pairs of patients with advanced NSCLC and their informal caregivers were recruited. The HRQoL of patients and informal caregivers was measured using the EORTC QLQ-C30 and the EQ-5D-5L scale, respectively. The structural equation modeling method was used to assess the pathways and potential mediating effects.

**Results:**

The average EQ-5D-5L score of informal caregivers was 0.92 ± 0.14. Caregiver occupation (*β* = −0.011, *p*=0.002), caregiving intensity (*β* = −0.013, *p*=0.017) and financial burden (*β* = −0.039, *p* < 0.001) were negatively associated with the HRQoL of informal caregivers. Overall, caregiving intensity was positively associated with financial burden (*β* = 0.242, *p* < 0.001) and health problems (*β* = 0.130, *p* < 0.001) among informal caregivers. Mediation analysis showed that financial burden (*β* = 0.021, *p*=0.016) and patient's HRQoL (*β* = 0.113, *p* < 0.001) were significant mediators in the association between caregiving intensity and caregivers' HRQoL.

**Conclusions:**

More efforts are required to reduce financial burden to diminish the negative impact of patient care needs on informal caregiver health. It is imperative to explore initiatives for enhancing support to informal caregivers.

## 1. Introduction

Lung cancer has the highest incidence and mortality rates globally, with 2.5 million new cases in 2022, accounting for 12.4% of the total new cases, and 1.8 million deaths due to lung cancer, representing 18.7% of total cancer deaths [1]. Non-small-cell lung cancer (NSCLC) comprised of approximately 80%–85% of all cases of lung cancer [2]. Approximately 70% of patients diagnosed with NSCLC are already in advanced stages [3], and the complex disease-related effects, high degree of disability, and expensive treatment costs cause significant psychological and physiological distress to patients, seriously affecting their quality of life and imposing a huge economic burden on the healthcare system [4].

Fragile health conditions and extensive side effects of treatment mean that patients with advanced NSCLC require long-term nursing support. Lung cancer patients have significantly higher supportive care needs than patients with any other cancer [5, 6]. In many cases, such support is provided by informal caregivers. Informal caregivers are usually family members, such as spouses, children, parents, relatives, etc., who deliver unpaid and ongoing care for the patient [7]. This care spans daily living assistance (e.g., feeding, bathing, dressing, and toileting), medical tasks (e.g., medication management), family issues (e.g., household activities), and emotional companionship [8].

In China, cultural norms rooted in familism emphasize mutual obligation, loyalty, and reciprocity among family members [9, 10]. As a result, informal caregiving is often assumed as a moral responsibility, deeply embedded in social expectations. Informal caregivers therefore play a pivotal role in supporting patients with chronic and terminal illnesses and have become an essential component of China's long-term care system, particularly amid population aging and the growing burden of cancers such as lung cancer.

While informal caregiving provides high-value contributions to both patients and the healthcare system, the consequences caregivers bear in doing so are substantial [11]. Current research suggests that informal caregiving sometimes serves as a beneficial experience or a demonstration of positivity [12, 13], but overall has a negative impact on the well-being of caregivers, or what is referred to as caregiver burden [14, 15]. Caregiver burden is commonly conceptualized as comprising both objective and subjective burden. Objective burden is the amount of time spent on caregiving and the number of tasks performed (i.e., caregiving intensity). Subjective burden encompasses caregivers' perceived physical, emotional, social, and financial distress related to their caregiving role [16, 17]. These emotional strains may include anxiety, sadness, or burnout; social burden may involve role conflicts, isolation, or disrupted family dynamics; while financial burden reflects the costs or reduced income resulting from caregiving responsibilities. Reviewing the literature, the multidimensional responses of informal caregivers due to caregiving burden have been well documented, including employment and financial security, physical and mental health, etc. [11, 18, 19]. Informal caregivers contribute to maintaining the well-being of lung cancer patients by providing emotional and practical support, often at great cost to their own quality of life [6, 20, 21].

To date, few studies have described both the objective and subjective caregiving burden that informal caregivers experience while caring for patients with advanced lung cancer. Informal caregivers, as the primary recipients of caregiving burden, have been the main focus of most empirical studies, which typically explore the impact of caregiving burden on their own health and well-being [8, 17]. However, limited attention has been paid to how these burdens may also influence patients' health outcomes, despite the interdependent nature of the caregiver-patient relationship. Reports that simultaneously examine both subjective and objective caregiving burden (e.g., caregiving intensity and financial burden), caregivers' health status, and patients' quality of life remain scarce. The caregiver-patient pair forms an interdependent unit, and understanding this dynamic is essential for informing interventions. Thus, to address this gap, the study focuses on informal caregiver-patient dyads, with the aims of examining how caregiving burden (including caregiving intensity and financial burden) relates to the health-related quality of life (HRQoL) of informal caregivers, as well as to explore their association with the HRQoL of patients with advanced lung cancer.

**Figure 1:**
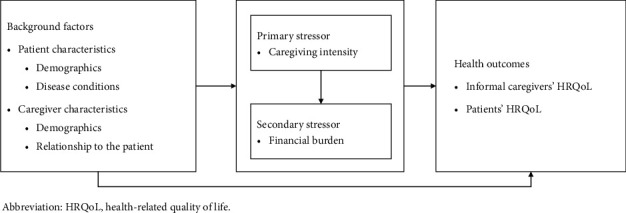
Research framework.

To better understand the complex mechanisms through which caregiving burden affects the health outcomes of informal caregivers and patients, we draw upon the Stress Process Model (SPM) [22], a widely used theoretical framework in chronic illness and caregiving research. The model conceptualizes caregiving as a dynamic and multistage process influenced by various factors, including the demographic backgrounds of both the patient and the informal caregiver, primary stressors (arising from core events or roles, such as caregiving intensity in this study), and secondary stressors (additional hardships derived from primary stressors, such as the family financial burden in this study). These stressors may ultimately impact the informal caregiver's health status through their cumulative effects. In this study, we extended this model to account for the interdependent relationship between informal caregivers and patients with advanced lung cancer, exploring how caregiving burden is not only associated with the caregivers' HRQoL but also with the HRQoL of the patients they care for (Figure 1). This framework provides a theoretical basis for investigating the mutual influences within the caregiver-patient relationship and underscores the necessity of comprehensive support strategies.

## 2. Materials and Methods

### 2.1. Design and Participants

This study is a part of the Demonstration Program on Health Technology Assessment, a nationwide investigation on the patients with locally advanced and metastatic NSCLC without sensitizing EGFR and ALK alterations in China [23]. We conducted this hospital-based cross-sectional study at 13 centers from November 2020 to June 2021. The enrolled participants were screened according to inclusion and exclusion criteria. The inclusion criteria for patients were (1) histologically or cytologically confirmed locally advanced or metastatic NSCLC (stage IIIB or IV) without EGFR/ALK mutation; (2) age ≥ 18 years; and (3) an ample level of physical and mental health to complete the questionnaire independently or with family assistance. Exclusion criteria were: (1) enrolment in clinical trials; (2) unable to understand the questionnaire; or (3) coexisting serious systemic diseases. Informal caregivers were recruited in parallel with patients. Inclusion criteria for caregivers were (1) family member of the patient, including spouse, children, parents, and other relatives; (2) age ≥ 18 years; (3) identified as nonpaid, primary caregiver, defined as the individual who provided the majority of care and support to the patient; and (4) able to understand and complete the questionnaire. Caregivers who were employed in a professional caregiving role (i.e., paid) were excluded.

### 2.2. Procedure

A set of structured questionnaires was independently designed for the target patients and informal caregivers, respectively, based on the literature review and expert opinion. Prior to the formal survey, we pretested the questionnaires and made adjustments and validations according to the pretest results. From November 2020 to June 2021, data were collected by face-to-face interviews using a combination of convenience sampling and cluster sampling in 13 centers in China, including eight general tertiary hospitals, three regional oncology hospitals, one pulmonary hospital, and one traditional Chinese medicine hospital from Jiangsu, Shanghai, Fujian, Shandong, and Sichuan provinces. For patients who were hospitalized multiple times during the survey period, each participant could only take the survey once. All surveys were conducted by trained researchers. Before the commencement of the survey, informed written consent was obtained from patients and their families, and the interviewer explained the questionnaire to each respondent to ensure they could clearly understand each question. Upon completion of each questionnaire, the interviewer conducted data monitoring to ensure data completeness and consistency.

### 2.3. Measures

The HRQoL of patients with advanced lung cancer was measured using the European Organization for Research and Treatment of Cancer (EORTC) Quality of Life Questionnaire (QLQ-C30) [24], which is one of the most widely used questionnaires for patient-reported outcomes in cancer research [25]. The most recent version of QLQ-C30 in use is version 3.0; its standardized Chinese version has been shown to be valuable in Chinese lung cancer populations [26]. The QLQ-C30 consists of 30 items, including a global health status/QoL scale, five functional subscales (physical, role, emotional, cognitive, and social), eight symptomatic subscales (fatigue, nausea/vomiting, pain, dyspnea, insomnia, appetite loss, constipation, and diarrhea), as well as the perceptions of financial difficulties resulting in the disease and treatment. With the exception of the two items of the global health status/QoL scale which had response options ranging from (1) “very poor” to (7) “excellent”, each of the remaining items had four response options: (1) “not at all”, (2) “a little”, (3) “quite a bit”, and (4) “very much”. The QLQ-C30 was scored according to a published scoring manual [27]. The raw scores for each domain and individual item were linearly converted to a value between 0 and 100. Higher scores indicate better health for functional scales and global health status/QoL scale, while lower scores represent more symptom burden or worse health for symptom scales. The QLQ-C30 summary score was calculated as the mean of the 13 subscales (excluding global health status/QoL scale and financial difficulties). Before calculating the mean, the symptom scales need to be reversed to obtain a uniform direction for all scales.

The European QoL-5 Dimensions (EQ-5D) scale is widely applied to measure HRQoL for general populations. We used the validated Chinese version of the five-level EQ-5D scale (EQ-5D-5L) to examine the HRQoL of informal caregivers. The EQ-5D-5L questionnaire consists of five dimensions (mobility, self-care, usual activities, pain/discomfort, and anxiety/depression), with a five-level response (1 = no problem, 2 = mild problem, 3 = moderate problem, 4 = severe problem, and 5 = unable/extreme problem). In this study, health utilities of informal caregivers were calculated using the Chinese population weights [28], which provide a range of scores from −0.391 to 1.000 (where 1 denotes perfect health, 0 denotes dead, and less than 0 denotes worse than dead).

Caregiving intensity was measured by the number of care hours provided by the informal caregiver per day [8, 22]. Four categories were used to capture the caregiving intensity from low to high, including up to 3 h, 3–6 h, 6–9 h, and more than 9 h. Informal caregivers were asked about the level of perceived financial burden on the family as a result of providing care, which was classified into three categories: mild, moderate, and severe.

In addition, basic demographic information was collected for both patients with advanced lung cancer and informal caregivers, including gender, age, education level (primary school and below, middle school, high school, and college and above), occupation (unemployed, civil servant, enterprise or institutional worker, freelancer, agriculture/forestry/livestock/fishery worker, retired, other), and relationship to the patient (spouse, parent, child, and other). For patients with advanced lung cancer, disease-related characteristics, clinical stage (IIIb, IIIc, IV), disease progression (yes and no), and months of disease duration (less than 6 months, 6–12 months, 12–24 months, more than 24 months) were also collected.

### 2.4. Statistical Analysis

Categorical variables are presented as frequencies and percentages, and continuous variables are presented as mean ± SD. Differences in caregiving intensity and financial burden among informal caregivers with different characteristics were examined by chi-square tests, and differences in HRQoL were examined by one-way ANOVA.

The HRQoL of patients with advanced lung cancer was measured using the QLQ-C30 scale, which involves three modules: global health status/QoL, symptoms, and functioning, along with the QLQ-C30 summary score. With reference to previous studies [29–31], the patients' HRQoL was represented by these four scores separately in subsequent analyses: Model 1 (patient's HRQoL as Global QoL), Model 2 (patient's HRQoL as functioning scales), Model 3 (patient's HRQoL as symptom scales), and Model 4 (patients' HRQoL as QLQ-C30 summary scores). Generalized linear regression was used to estimate the factors influencing the HRQoL of patients with advanced NSCLC and their informal caregivers.

**Figure 2:**
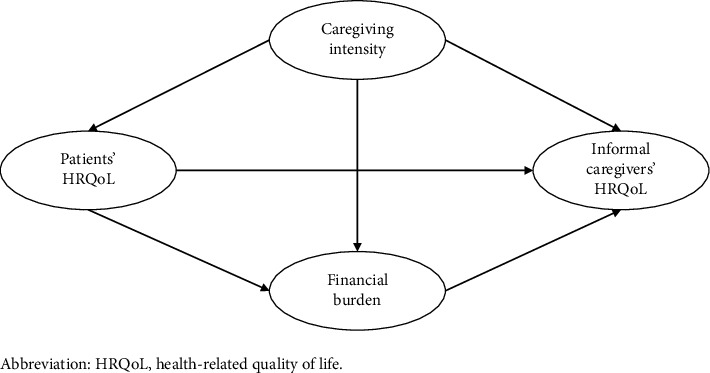
Analytical framework of structural equation modeling.

In addition, structural equation modeling (SEM) was used to determine the relationship and mechanisms between caregiving intensity, family financial burden, and HRQoL among patients and informal caregivers, and to examine the potential mediating effects. The analytical framework is shown in Figure 2. The following criteria were used for assessing the fitness of the model: *χ*^2^/df ratio < 5, comparative fit index (CFI) and Tucker–Lewis Index (TLI) > 0.85, and root mean square error of approximation (RMSEA) and standardized root mean square residual (SRMR) < 0.08 [32]. Parameters were estimated using maximum likelihood. It is noted that caregivers' HRQoL was a latent variable constructed on the five dimensions of the EQ-5D scale in the path analysis, with higher scores indicating more severe health problems.

Statistical significance was defined as *p* < 0.05 [33]. All analyses were performed using Mplus 7.0.

## 3. Results

### 3.1. Sample Characteristics

In total, 540 patients with advanced lung cancer and their corresponding 540 informal caregivers were included in the study. The patients were predominantly male (77.41%) with a mean age of 64.01 ± 9.39 years. Informal caregivers were predominantly female (58.33%), with a mean age of 50.09 ± 13.08 years. The majority (37.59%) spent over 9 h per day on caregiving, and 43.33% perceived a heavy financial burden on their families.  Table 1 demonstrates the characteristics of the study sample. According to Table 2, there was a significant difference in caregiving intensity as well as financial burden among informal caregivers with different household registration, education level, occupation, and relationship with the patient (*p* < 0.05).

### 3.2. HRQoL and the Determinants

Table 3 shows that the percentages of informal caregivers who have no problems on the five dimensions of mobility, self-care, usual activities, pain/discomfort, and anxiety/depression are 87.59%, 93.15%, 87.96%, 74.07%, and 54.07%, respectively. Among the five dimensions, the anxiety/depression dimension had the highest number and severity of problems. The mean score of health utility of informal caregivers was 0.918 ± 0.145. The results of the variance analysis indicated that longer daily caregiving hours and heavier financial burdens were associated with lower health utilities among informal caregivers (Table 2). The mean value of the QLQ-C30 summary score for patients with advanced lung cancer was 80.70 ± 14.94. Specifically, in the functional subscales, social functioning scored the highest, followed by emotional functioning, while physical functioning scored the lowest. In the symptom subscales, financial difficulties scored the highest, followed by fatigue symptoms, with diarrhea symptoms scoring the lowest. Supporting Table 1 displays the distribution and scores of HRQoL of patients.

The impacts of patient-level and caregiver-level factors on the QoL of patients and informal caregivers were presented in Table 4. For informal caregivers, patient's occupation, caregiver's occupation, caregiving intensity, and the financial burden significantly affected their QoL. For patients with advanced lung cancer, regardless of scales, both caregiving intensity and financial burden were significantly negatively associated with patients' HRQoL (*p* < 0.05). In addition, patient's occupation, clinical stage, and disease progression also almost universally impact patients' HRQoL significantly (*p* < 0.05). Specifically, the closer the clinical stage to the advanced stage and the presence of disease progression, the worse the HRQoL for patients.

### 3.3. Path Analyses

Table 5 reveals the complex relationship between caregiving intensity, financial burden, QoL of informal caregivers, and HRQoL of patients with advanced lung cancer. The fitness of each model met the recommended criteria (see Supporting Table 2). In Model 1, caregiving intensity was inversely related to patient's global QoL (*β* = −0.107, *p*=0.015), but directly proportional to the informal caregiver's health problems (*β* = 0.088, *p*=0.020) and financial burden (*β* = 0.229, *p* < 0.001). Additionally, financial burden mediates the path from caregiving intensity to QoL of informal caregivers (*β* = 0.021, *p*=0.016). An increase in patient's Global QoL score can positively influence the reduction of informal caregivers' health problems by alleviating the financial burden (*β* = −0.011, *p*=0.048). In the overall pathway of caregiving intensity's influence on the HRQoL of informal caregivers, the mediating effect played by financial burden accounted for 16.15%. In Model 2, improved patient functioning contributed to a lower risk of health problems for informal caregivers (*β* = −0.293, *p* < 0.001) and also reduced family financial burden (*β* = −0.011, *p*=0.048). In addition, better patient outcomes mediated the effect of caregiving intensity on financial burden (*β* = 0.053, *p*=0.004). The mediating effect played by financial burden accounted for 10.77% of the total pathway of the effect of caregiving intensity on the HRQoL of informal caregivers. The similar pattern was found in Model 3. The higher the symptom score of patients, the more likely informal caregivers were to have health problems (*β* = 0.340, *p* ≤ 0.001). In Model 4, caregiving intensity significantly increased QLQ-C30 summary scores of patients (*β* = 0.343, *p* < 0.001) and family financial burden (*β* = 0.189, *p* < 0.001). There was a positive association between QLQ-C30 summary scores and poor health of informal caregivers (*β* = 0.330, *p* < 0.001) and financial burden (*β* = 0.155, *p*=0.001). In addition, caregiving intensity through patient's HRQoL mediated not only informal caregiver' health (*β* = 0.113, *p* < 0.001) but also financial burden (*β* = 0.053, *p*=0.002). The mediating effect played by financial burden accounted for 10.00%.

## 4. Discussion

To the best of our knowledge, this is the first study to investigate both subjective and objective caregiving burdens (caregiving intensity and financial burden) and their relationships with QoL among informal caregivers of patients with advanced lung cancer. Our findings add to the current literature by not only confirming direct associations but also by unveiling complex pathways of interaction among these variables and identifying crucial mediating effects. Additionally, by applying and extending SPM, our study enhances the theoretical understanding of informal caregiving dynamics and offers a foundation for future interventions aimed at supporting caregiving dyads in oncology settings.

Overall, we found detrimental effects of caregiving burden on the health of informal caregivers, which is consistent with many previous studies. A moderate to high level of caregiving intensity was observed in nearly 70% of informal caregivers in our study sample, with 37.59% spending at least 63 h of care per week. This intensity far exceeds the common threshold of 20 h per week, often used in previous studies to identify low-intensity care regardless of specific circumstances [34]. Individuals providing over 20 h of care per week have been associated with a 20% higher likelihood of experiencing mental health problems [35], underscoring caregiving intensity as a significant determinant of mental health challenges [36]. Patients with advanced lung cancer often suffer from a variety of symptoms simultaneously related to both the primary disease itself and its demanding treatment. During this period, patients may frequently undergo invasive and toxic therapies, and the symptoms caused by lung cancer are reported to be the most intense across cancer types [6], severely impairing their QoL. Highly prevalent respiratory system symptoms like dyspnea, cough, and hemoptysis can cause significant distress in caregiving [6]. The complexity of these caregiving tasks requires substantial time commitment, and the patient's suffering is mapped onto the caregiver's physical strain and emotional demands. Furthermore, extensive caregiving duties may restrict caregivers' participation in essential social activities [37], potentially exacerbating isolation and burden.

Crucially, our findings illuminate the deeply symbiotic relationship between informal caregivers and patients with advanced lung cancer, a novel contribution to the literature. We demonstrate that each deterioration in a patient's health represents an additional drain on the informal caregiver's QoL, extending beyond mere correlation to a dynamic interplay. In most cases, the worse the patient's health, the greater the time and effort demanded for care, often at the expense of the informal caregiver's own well-being, leading to exacerbation of their health problems. Conversely, a higher QoL for the patient is associated with a lower risk of caregiver health problems arising from their caregiving role. Unlike paid institutional caregivers, family members in informal care roles are inherently more attuned to the patient's profound suffering [6]. Moreover, caregivers may experience profound sadness and a sense of powerlessness as the disease progresses, which further contributing to the development of depression or anxiety. This highlights that patient well-being is not merely an outcome in itself but a crucial determinant of caregiver health, establishing a direct pathway of influence from patient health to caregiver QoL.

In addition, our study uniquely identified significant mediating effects within the caregiving burden model. Specifically, financial burden played a substantial mediating role, accounting for over 16% of the total effect of caregiving intensity on caregiver's QoL. This suggests that the time commitment required by intense caregiving directly or indirectly leads to economic difficulties, which in turn substantially erodes the caregiver's QoL. Therefore, interventions aimed at alleviating the financial pressures on informal caregivers could potentially mitigate a substantial portion of the negative health consequences associated with high-intensity caregiving. Besides, the moderating role of financial burden was also been demonstrated in the pathway from patient health to informal caregiver health. For instance, even though the QoL of informal caregivers and patients is intertwined, approaches can be found to diminish the adverse effects of poor patient outcomes on informal caregiver health by reducing the financial burden resulting from caregiving. This finding highlights financial support is not just a consequence but an active mediator of the caregiving experience. Considering the negative impact of financial burdens on the health and overall well-being of informal caregivers, the possibility of informal caregivers being caught in a vicious cycle of illness-induced poverty and poverty-induced illness due to caregiving warrants the attention from society and policymakers. These intricate relationships underscore the necessity of a holistic support approach that considers both direct care demands and the cascading financial consequences to truly improve outcomes for both caregivers and patients.

### 4.1. Limitations

This study has some limitations. First, the cross-sectional investigation does not allow us to propose causal relationships. However, for the population of patients with advanced cancer, the larger sample size and the exploratory nature of this study permits us to generate new hypotheses for future research. Second, we surveyed patients who were recipients of informal caregiving, but we were unable to identify those receiving mixed care. Third, all data collected in this study rely on self-reporting, which is prone to bias. Additionally, detailed clinical characteristics of patients, such as metastasis status and comorbidities, were not collected in this study. These factors may influence caregivers' HRQoL and should be considered in future research to enhance the comprehensiveness and validity of the findings. More robust results are expected in the future by introducing some objective measures.

### 4.2. Practice Implications

Research has shown that formal care resources are helpful, but the cost of such resources will rise as the population ages [38]. Under the context of scarce care resources and rising demand for care, informal care has become an important component of healthcare systems to remain sustainable and affordable in the future. In China, the contribution of informal caregivers in providing care rarely receives recognition and support, although Chinese population rely more on home-based informal care than on institutionalized care. Providing care for family members seems to be a socially default norm of family obligation. However, it is important to emphasize that not only developed countries [35] but also China are facing declining family size, fewer intergenerational households, and increasing female labor force participation. In other words, the availability of informal caregivers is decreasing. Paradoxical developments imply that the burden on informal caregivers will increase in the future. To mitigate the negative impact of caregiving burden on informal caregivers and to sustain an adequate pool of healthy informal caregivers for the future, thereby improving the QoL of patients and promoting societal well-being, it is necessary to provide relevant support for informal caregivers. Previous studies have noted that coping skills and mechanisms and social support can reduce the negative impact of the caregiving role, as it acts as a buffer to lessen the impact of caregiving stressors on the caregiver [39]. The establishment of a systematic support network for informal caregivers can shorten the time needed for caregiving and reduce the caregiving burden. Services may support in several ways, including training, counselling and infrastructure strengthening [40], and linking informal caregivers with health professionals and social workers to enable them to become skilled in caring for their patients. Some studies have suggested that family caregivers' coping strategies can help them regulate emotions [24].

## 5. Conclusions

In conclusion, our study reveals significant associations between the subjective and objective caregiving burden of informal caregivers of patients with advanced NSCLC and their QoL. Caregiving intensity significantly increased caregivers' financial burden and risk of health problems, and financial burden and patient QoL mediated caregiver QoL. These findings offer a novel target for intervention, potentially breaking the vicious cycle of illness and poverty for these vulnerable caregivers and highlighting an essential area for societal attention and support.

## Figures and Tables

**Table 1:** Characteristics of patients with advanced lung cancer and informal caregivers.

Variables	Patients with advanced lung cancer (*N* = 540)	Informal caregivers (*N* = 540)
	*n* (%)	*n* (%)
Gender		
Female	122 (22.59)	315 (58.33)
Male	418 (77.41)	225 (41.67)
Age	64.01 ± 9.39	50.09 ± 13.08
Education level		
Primary school and below	201 (37.22)	112 (20.74)
Middle school	183 (33.89)	162 (30.00)
High school	115 (21.30)	147 (27.22)
College and above	41 (7.59)	119 (22.04)
Occupation		
Unemployed	100 (18.52)	70 (12.96)
Civil servant	8 (1.48)	9 (1.67)
Enterprise or institutional employee	44 (8.15)	77 (14.26)
Freelancer	61 (11.30)	125 (23.15)
Agriculture/Forestry/Livestock/Fishery worker	89 (16.48)	77 (14.26)
Retired	175 (32.41)	99 (18.33)
Other	63 (11.67)	83 (15.37)
Clinical stage		
IIIb	150 (27.78)	—
IIIc	33 (6.11)	—
IV	357 (66.11)	—
Disease progression		
No	325 (60.19)	—
Yes	215 (39.81)	—
Course of disease		
Up to 6 months	222 (41.11)	—
6–12 months	118 (21.85)	—
12–24 months	94 (17.41)	—
More than 24 months	106 (19.63)	—
Relationship to the patient		
Spouse	—	266 (49.26)
Parent	—	30 (5.56)
Child	—	219 (40.56)
Other	—	25 (4.63)
Caregiving intensity/Daily care time		
Up to 3 h	—	164 (30.37)
3–6 h	—	126 (23.33)
6–9 h	—	47 (8.70)
More than 9 h	—	203 (37.59)
Financial burden		
Mild	—	57 (10.56)
Moderate	—	249 (46.11)
Severe	—	234 (43.33)

**Table 2:** Univariate analysis of caregiving intensity, financial burden and the HRQoL of informal caregivers.

	Caregiving intensity					Financial burden				HRQoL	
	Up to 3 h	3–6 h	6–9 h	More than 9 h	*p*	Mild	Moderate	Severe	*p*	Mean (SD)	*p*
	*n* (%)	*n* (%)	*n* (%)	*n* (%)		*n* (%)	*n* (%)	*n* (%)			
Gender					0.002				0.19		0.98
Female	84 (26.67)	65 (20.63)	26 (8.25)	140 (44.44)		29 (9.21)	140 (44.44)	146 (46.35)		0.919 (0.122)	
Male	80 (35.56)	61 (27.11)	21 (9.33)	63 (28.00)		28 (12.44)	109 (48.44)	88 (39.11)		0.918 (0.172)	
Education level					< 0.001				< 0.001		0.09
Primary school and below	13 (11.61)	22 (19.64)	9 (8.04)	68 (60.71)		4 (3.67)	52 (46.43)	56 (50.00)		0.893 (0.124)	
Middle school	35 (21.60)	41 (25.31)	12 (7.41)	74 (45.68)		17 (10.49)	54 (33.33)	91 (56.17)		0.922 (0.157)	
High school	60 (40.82)	36 (24.49)	13 (8.84)	38 (25.85)		18 (12.24)	73 (49.66)	56 (38.10)		0.938 (0.136)	
College and above	56 (47.06)	27 (22.69)	13 (10.92)	23 (19.33)		18 (15.13)	70 (58.82)	31 (26.05)		0.913 (0.153)	
Occupation					< 0.001				0.002		0.38
Unemployed	13 (18.57)	11 (15.71)	8 (11.43)	38 (54.29)		2 (2.86)	25 (35.71)	43 (61.43)		0.935 (0.084)	
Civil servant	3 (33.33)	3 (33.33)	1 (11.11)	2 (22.22)		2 (22.22)	5 (55.65)	2 (22.22)		0.960 (0.054)	
Enterprise or institutional employee	33 (42.86)	17 (22.08)	8 (10.39)	19 (24.68)		14 (18.18)	39 (50.65)	24 (31.17)		0.933 (0.134)	
Freelancer	45 (36.00)	34 (27.20)	12 (9.60)	34 (27.20)		9 (7.20)	61 (48.80)	55 (44.00)		0.929 (0.130)	
Agriculture/Forestry/Livestock/Fishery worker	18 (23.38)	22 (28.57)	6 (7.79)	31 (40.26)		5 (6.49)	34 (44.16)	38 (49.35)		0.911 (0.111)	
Retired	18 (18,18)	18 (18.18)	8 (8.08)	55 (55.56)		17 (17.17)	40 (40.40)	42 (42.42)		0.897 (0.186)	
Other	34 (40.96)	21 (25.30)	4 (4.82)	24 (28.92)		8 (9.64)	45 (54.22)	30 (36.14)		0.903 (0.186)	
Relationship to the patient					< 0.001				0.03		< 0.001
Spouse	52 (19.55)	44 (16.54)	24 (9.02)	146 (54.89)		26 (9.77)	100 (40.60)	132 (49.62)		0.900 (0.148)	
Parent	10 (33.33)	10 (33.33)	2 (6.67)	8 (26.67)		3 (10.00)	10 (33.33)	17 (56.67)		0.948 (0.088)	
Child	91 (41.55)	63 (28.77)	21 (9.59)	44 (20.09)		25 (11.42)	117 (53.42)	77 (35.16)		0.848 (0.326)	
Other	11 (44.00)	9 (36.00)	0 (0.00)	5 (20.00)		3 (12.00)	14 (56.00)	8 (32.00)		0.939 (0.109)	
Caregiving intensity					—				< 0.001		0.003
Up to 3 h	—	—	—	—		24 (14.63)	98 (59.76)	42 (25.61)		0.943 (0.087)	
3–6 h	—	—	—	—		6 (4.76)	73 (57.94)	47 (37.30)		0.935 (0.103)	
6–9 h	—	—	—	—		8 (17.02)	16 (34.04)	23 (48.94)		0.900 (0.162)	
More than 9 h	—	—	—	—		19 (9.36)	62 (30.54)	122 (60.10)		0.892 (0.189)	
Financial burden					—				—		< 0.001
Mild	—	—	—	—		—	—	—		0.970 (0.055)	
Moderate	—	—	—	—		—	—	—		0.933 (0.105)	
Severe	—	—	—	—		—	—	—		0.891 (0.186)	

Abbreviation: HRQoL, health-related quality of life.

**Table 3:** The distributions and scores of EQ-5D-5L in informal caregivers.

Health problems	Mobility	Self-care	Usual activities	Pain/discomfort	Anxiety/depression
Having no problems (*n* (%))	473 (87.59)	503 (93.15)	475 (87.96)	400 (74.07)	292 (54.07)
Having slight problems (*n* (%))	52 (9.63)	28 (5.19)	51 (9.44)	112 (20.74)	175 (32.41)
Having moderate problems (*n* (%))	11 (2.04)	6 (1.11)	7 (1.30)	20 (3.70)	57 (10.56)
Having severe problems (*n* (%))	2 (0.37)	2 (0.37)	5 (0.93)	5 (0.93)	11 (2.04)
Having extreme problems (*n* (%))	2 (0.37)	1 (0.19)	2 (0.37)	3 (0.56)	5 (0.93)
Health utility values (*n* (%))	0.012 (0.039)	0.005 (0.023)	0.008 (0.029)	0.021 (0.045)	0.035 (0.051)
Health utilities (mean, SD)	0.918 (0.145)				

*Note:* EQ-5D-5L, five-level EQ-5D scale.

**Table 4:** Regression analysis of the influencing factors of the HRQoL of patients with advanced lung cancer and informal caregivers.

Variables	HRQoL of patients with advanced lung cancer								HRQoL of informal caregivers	
	Model 1		Model 2		Model 3		Model 4		*β*	*p*
	*β*	*p*	*β*	*p*	*β*	*p*	*β*	*p*		
Patients with advanced lung cancer										
Gender	−0.061	0.2	0.046	0.3	−0.055	0.22	0.052	0.25	0.030	0.53
Age	−0.101	0.05	−0.043	0.37	0.028	0.56	−0.048	0.32	−0.039	0.44
Education	0.030	0.57	0.082	0.09	−0.088	0.07	0.086	0.08	−0.028	0.58
Occupation	0.099	0.04	0.119	0.007	−0.085	0.05	0.102	0.02	0.104	0.02
Clinical staging	0.003	0.94	−0.084	0.04	0.088	0.03	−0.080	0.05	−0.048	0.27
Disease progression	−0.104	0.08	−0.172	0.002	0.170	0.002	−0.182	< 0.001	−0.062	0.28
Course of disease	0.104	0.07	0.072	0.18	−0.082	0.12	0.086	0.11	0.044	0.44
Informal caregivers										
Gender	−0.089	0.06	0.012	0.79	−0.005	0.92	0.014	0.75	−0.028	0.56
Age	0.058	0.35	−0.043	0.46	0.078	0.18	−0.066	0.26	−0.065	0.28
Education	−0.102	0.08	−0.070	0.2	0.057	0.29	−0.077	0.16	−0.035	0.54
Occupation	−0.042	0.36	−0.064	0.14	0.041	0.34	−0.058	0.19	−0.140	0.002
Relationship to the patient	0.052	0.34	−0.037	0.47	0.050	0.33	−0.052	0.31	0.008	0.88
Caregiving intensity	−0.110	0.02	−0.287	< 0.001	0.284	< 0.001	−0.287	< 0.001	−0.105	0.03
Financial burden	−0.147	< 0.001	−0.146	< 0.001	0.203	< 0.001	−0.145	< 0.001	−0.175	< 0.001

Abbreviation: HRQoL, health-related quality of life.

**Table 5:** Pathway analysis of caregiving intensity, financial burden, and the HRQoL of informal caregivers and patients with advanced lung cancer.

Paths	Model 1		Model 2		Model 3		Model 4	
	*β*	*p*	*β*	*p*	*β*	*p*	*β*	*p*
Direct effects								
Caregiving intensity ⟶ patients' HRQoL	−0.107	0.02	−0.342	< 0.001	0.325	< 0.001	0.343	< 0.001
Caregiving intensity ⟶ informal caregivers' HRQoL	0.088	0.02	0.012	0.79	0.002	0.96	0.001	0.99
Caregiving intensity ⟶ financial burden	0.229	< 0.001	0.189	< 0.001	0.196	< 0.001	0.189	< 0.001
Patients' HRQoL ⟶ informal caregivers' HRQoL	−0.186	0.01	−0.293	< 0.001	0.340	< 0.001	0.330	< 0.001
Patients' HRQoL ⟶ financial burden	−0.122	0.009	−0.154	0.002	0.141	0.003	0.155	< 0.001
Financial burden ⟶ informal caregivers' HRQoL	0.091	0.01	0.072	0.04	0.069	0.05	0.067	0.066
Indirect effects								
Caregiving intensity ⟶ informal caregivers' HRQoL	0.042	0.007	0.118	< 0.001	0.127	< 0.001	0.129	< 0.001
Caregiving intensity ⟶ patients' HRQoL ⟶ informal caregivers' HRQoL	0.020	0.11	0.100	< 0.001	0.111	< 0.001	0.113	< 0.001
Caregiving intensity ⟶ financial burden ⟶ informal caregivers' HRQoL	0.021	0.02	0.014	0.08	0.014	0.09	0.013	0.10
The mediating effect proportion of financial burden	16.15%	—	10.77%	—	10.77%	—	10.00%	—
Caregiving intensity ⟶ Patients' HRQoL ⟶ financial burden	0.013	0.07	0.053	0.004	0.046	0.006	0.053	0.002
Patients' HRQoL ⟶ financial burden ⟶ informal caregivers' HRQoL	−0.011	0.04	−0.011	0.07	0.010	0.09	0.010	0.09
Total effects								
Caregiving intensity ⟶ patients' HRQoL	−0.107	0.02	−0.342	< 0.001	0.325	< 0.001	0.343	< 0.001
Caregiving intensity ⟶ informal caregivers' HRQoL	0.130	< 0.001	0.130	< 0.001	0.130	< 0.001	0.130	< 0.001
Caregiving intensity ⟶ financial burden	0.242	0.01	0.242	< 0.001	0.242	< 0.001	0.242	< 0.001
Financial burden ⟶ informal caregivers' HRQoL	0.091	0.01	0.072	0.04	0.069	0.06	0.067	0.07

Abbreviation: HRQoL, health-related quality of life.

## Data Availability

The informed consent for participants stated that the data was used for this study only and the dataset involved the privacy of participants from multiple institutions. Maybe we cannot share participants data at this time. Requests to access the datasets should be directed to yychen@shmu.edu.cn.

## Supporting Information

Supporting Table 1. The scores of EORTC QLQ-C30 in patients with advanced NSCLC.

Supporting Table 2. The fitness of the model.
