# Supplementary material for: The Impact of Caregiving Intensity and Financial Burden on the Health-Related Quality of Life Among Informal Caregivers for Patients With Advanced Lung Cancer: A Multicenter Cross-Sectional Study
Source: J Nurs Manag. 2025 Oct 30;2025:8847157. doi: 10.1155/jonm/8847157 (PMC12591805; doi:10.1155/jonm/8847157)
Supplement: Supporting Information — Additional supporting information can be found online in the Supporting Information section. [file 8847157.f1.docx]

**Supplementary Table 1.** The scores of EORTC QLQ-C30 in patients with advanced NSCLC

| Scale | Mean (SD) |
| --- | --- |
| Global health status/QoL scale | 62.95 (19.06) |
| Functional scales |  |
| Physical functioning | 76.99 (20.93) |
| Role functioning | 80.02 (24.11) |
| Emotional functioning | 81.38 (19.35) |
| Cognitive functioning | 69.94 (27.43) |
| Social functioning | 83.40 (19.26) |
| Symptom scales |  |
| Fatigue | 26.93 (22.40) |
| Nausea and vomiting | 20.62 (22.72) |
| Pain | 10.24 (17.03) |
| Dyspnoea | 22.01 (23.69) |
| Insomnia | 22.35 (26.07) |
| Appetite loss | 22.04 (25.36) |
| Constipation | 12.41 (19.90) |
| Diarrhoea | 7.37 (16.57) |
| Financial difficulties | 42.64 (34.20) |
| QLQ-C30 summary score | 80.70 (14.94) |

**Supplementary Table 2.** The fitness of the model

|  | χ^2^/df | CFI | TLI | RMSEA | SRMR |
| --- | --- | --- | --- | --- | --- |
| Model 1 | 4.194 | 0.958 | 0.931 | 0.079 | 0.044 |
| Model 2 | 5.326 | 0.916 | 0.890 | 0.092 | 0.056 |
| Model 3 | 3.379 | 0.923 | 0.906 | 0.068 | 0.048 |
| Model 4 | 3.619 | 0.903 | 0.888 | 0.072 | 0.050 |
